# Impact of COVID-19 Response Strategies on Frontline Health Workers' Motivation: A Case Study in Two Regional Referral Hospitals in Ghana and Uganda

**DOI:** 10.24248/eahrj.v8i1.761

**Published:** 2024-03-28

**Authors:** Simon Peter Katongole, Peter Badimak Yaro, Paul Bukuluki

**Affiliations:** a School of Public Health, Gudie University Project; b Basic Needs Ghana; c Department of Social Work and Social Administration, Makerere University, Uganda

## Abstract

**Background:**

Several health systems developed interventions and strategies in response to the COVID-19 pandemic, some of which were broad-based, some of which focused on service delivery, and others on frontline health workers. The goal of this study was to see how COVID-19 interventions affected the motivation of frontline health workers in Ghana and Uganda.

**Methods:**

The research was undertaken during the period of May to July 2020, coinciding with the initial three months of the global response to the COVID-19 pandemic. This was a critical juncture when the majority of the proposed strategies were in the nascent stages of execution. The research methodology employed was cross-sectional study design, utilizing a qualitative phenomenological approach. The study was conducted across two regional referral hospitals located in Ghana and Uganda. Sixteen frontline healthcare workers from both Uganda and Ghana were selected for interviews, which were conducted both in-person and telephonically. Additionally, one managerial staff member from each hospital was also interviewed. The collected data were subsequently subjected to a deductive thematic analysis.

**Results:**

The three themes that emerged from the study include the interventions mentioned by the healthcare workers, the interventions that serve as motivators, and those interventions that act as demotivators. The conceptualization and implementation of the COVID-19 response interventions resulted in differential perceptions regarding their impact on the motivation of healthcare workers in the two hospitals under study. The primary catalysts for healthcare professionals' motivation were the leadership exhibited by their respective governments and supervisors, coupled with certain promises made. These included financial incentives that initially appeared to be motivational, as well as proposals to offer complimentary services. However, the failure to fulfill some of these commitments, along with the unequal distribution of the financial incentives, led to a decrease in motivation among the healthcare workers. Certain human resources for health strategies were perceived as poorly planned and impracticable, resulting in a demotivation among COVID-19 frontline healthcare providers.

**Conclusion:**

The COVID-19 response interventions' implementation yielded varied perceptions on healthcare workers' motivation both positive and negative. These were attributed to leadership quality, promises like financial incentives and complementary services, and unmet commitments. The COVID-19 pandemic response strategies in both countries underscore the need for preparedness in the face of unforeseen outbreaks. Failure to sustain healthcare worker motivation may compromise future response effectiveness. Governments must learn from this and come up with well sought of response strategies incorporating health workers' input for comprehensive crisis management. They should establish well-resourced, multisectoral units with specific incentives to handle the response.

## BACKGROUND

The advent of the novel Coronavirus 19 (COVID-19) posed a significant challenge to various health systems worldwide, which found themselves grappling with the task of formulating an effective response to the pandemic. This situation necessitated the development and implementation of diverse strategies aimed at enhancing and fortifying their capacity to respond to such an epidemic. These strategies, tailored to address the COVID-19 pandemic, were designed with the dual objectives of ensuring the preservation of public health at a population level, and facilitating the efficient management of COVID-19 patients across different levels of the healthcare system. A number of countries formulated their response plans by drawing upon the experiences and lessons gleaned from China, the country that reported the initial cases of the virus on a global scale^1^. In the wake of the COVID-19 pandemic, other various nations were compelled to draw upon the knowledge acquired from the SARS epidemic to formulate their response strategies^2^. Nonetheless, the implementation of these strategies may have engendered a spectrum of hitherto unexamined experiences, outcomes, and perceptions. This is particularly pertinent to healthcare professionals, who bear the primary responsibility for executing these plans, especially within the hospital setting.

The advent of COVID-19 in Africa was delayed relative to Western, Asian, American, and other global regions. The necessity of ensuring the safety and health of healthcare workers for the successful implementation of response plans aimed at managing the COVID-19 pandemic had been emphasized.^3^ As a result, the majority of African nations formulated their response strategies based on the measures they observed being implemented in countries where the pandemic had previously been reported.^4^ Furthermore, numerous African countries drew upon their experiences with the Ebola outbreak to inform their COVID-19 response strategies.^5^ At the level of healthcare systems, policy and response plans were designed to address either the delivery of health services or the improvement and maintenance of the welfare of health personnel, particularly those on the front lines of the pandemic. Given their crucial role in managing the pandemic, the care and motivation of health workers were prioritized in the response plans.^6^

The implementation of response strategies ostensibly played a pivotal role in mitigating the repercussions of the COVID-19 pandemic. However, these strategies also had profound implications on the delivery of health services and the health workers at the forefront of providing care to COVID-19 patients. While certain measures were primarily aimed at minimizing the risk of infection among health workers through Infection Prevention Control (IPC) and Standard Operating Procedures (SOPs), the effectiveness of these risk mitigation strategies is largely contingent upon the motivation of the health workers. Perceptions of health workers towards these response plans, particularly if they believe that the plans are not implemented in good faith, can significantly affect their motivation. Consequently, this could have a detrimental effect on the adherence to these risk mitigation measures^6^.

The efficacy and success of the measures implemented to combat the COVID-19 pandemic are intrinsically linked to their impact on the motivation of healthcare workers. Therefore, it was of paramount importance to examine the perceptions of the healthcare workers towards the strategies employed, especially in terms of their motivational implications. This examination could provide a basis for the reconfiguration and fortification of response measures, taking into account their influence on the motivation of healthcare workers to respond effectively. However, there is a paucity of knowledge regarding how the experiences of healthcare workers with these measures have shaped their motivation to respond to the pandemic in the context of Sub-Saharan Africa. Utilizing a phenomenological approach and case studies from Ghana and Uganda, we elucidate the perceptions of healthcare workers towards the plans, policies, and measures enacted in response to COVID-19, and how these perceptions have influenced their motivation to deliver patient care services.

## METHODS

### Study Design

This qualitative study, conducted from May to July 2020, three months into the COVID-19 pandemic, employed a phenomenological methodology to explore the perceptions and viewpoints of frontline health workers regarding the motivational impact of COVID-19 response strategies and policies. Recognizing the experiences of frontline health workers was deemed crucial in the COVID-19 context for the development of suitable responses catering to both health workers and patients.^7^ Phenomenology is also considered instrumental in delving into the depth of individuals' lived experiences, beliefs, and perceptions.^8^ Semi-structured interviews featuring open-ended dialogue were utilized to achieve a more profound and extensive comprehension of health workers' experiences resulting from the implementation of policies and guidelines to control and manage the COVID-19 pandemic.

### Study Setting

The research was conducted in two purposively chosen regional referral hospitals in Ghana and Uganda, due to their significant role as primary COVID-19 treatment centers during the initial stages of the pandemic in their respective regions. The Tamale Teaching Hospital (TTH), serving as the Northern Regional Referral Hospital in Ghana, has provided treatment for COVID-19 patients from Burkina Faso, Togo and Ivory Coast.

In Uganda, the study was undertaken at the Entebbe Regional Referral Hospital (ERRH), which is situated in close proximity to the Entebbe International Airport. ERRH serves a dual purpose: it is the primary healthcare entry point for air travelers entering Uganda, and it also functions as a referral hospital for Wakiso, the second most populous district in Uganda after the capital, Kampala. Wakiso uniquely surrounds Kampala, the capital city of Uganda.

Given the initial perception of COVID-19 as an imported disease, the Ugandan government designated ERRH, along with the national referral hospital, as a key institution in the management of COVID-19 patients. This decision was based on the view of ERRH as the primary entry point for COVID-19 patients arriving via air travel.

### Study Participants

The study participants were health workers who were integral members of the teams designated to deliver services to patients diagnosed with COVID-19 at the onset of the pandemic. Thirteen health workers, who willingly consented to participate, were interviewed from two distinct hospitals: seven from Entebbe Regional Referral Hospital (ERRH) and six from Tamale Teaching Hospital (TTH) located in the Northern region of Ghana. The cohort from TTH comprised of three nurses and three medical officers, while the ERRH group consisted of two nurses, three medical officers, one hygienist, and one ambulance driver. The selection of these health workers was based on a convenience sampling strategy, taking into account their specific roles in the COVID-19 response and the feasibility of contacting them due to the restrictions implemented to mitigate the spread of COVID-19 during the initial phase of the pandemic.

### Data Collection

Interviews of a semi-structured nature were undertaken, utilizing both remote and in-person methodologies as dictated by the prevailing circumstances. In Uganda, stringent lockdown measures imposed on frontline health workers necessitated the execution of six interviews via telephone. Nevertheless, an in-person interview was facilitated with a medical officer in tranquil and secure setting post-work hours. In contrast, all of the interviews in TTH were conducted face to face because it was physically easier to reach out to the interviewees. In compliance with COVID-19 standard operating procedures including the wearing of face masks/shields, a four-meter distance was maintained between the interviewer and interviewee during all in-person interviews. All interviews at TTH were conducted by PBY. The medium of communication for all interviews was English, facilitated by an interview guide encompassing discussion topics. With the consent of the participants, all interviews were audio recorded. The interview process was continued until data saturation was achieved in each country, i.e., no new information was forthcoming.^9^

### Data Management and Analysis

The interviews were transcribed verbatim in English by SPK. Each of the three authors independently scrutinized the data, repeatedly perusing the transcripts to establish codes, sub-themes, and major themes, utilizing the inductive thematic analysis framework.^10–12^ Upon completion of their individual review and analysis, the authors exchanged their notes for re-reading to achieve a consensus. Subsequently, a virtual meeting was convened by all three authors to reconcile and agree on the themes derived from their individual analyses. The findings are elucidated with the inclusion of pertinent quotes as necessary.

### Ethics Approval and Informed Consent

The study was conducted under the auspices of the Mildmay Uganda Research Ethics Committee (MUREC), which granted approval (REC REF 0706-2021). This ethical clearance was also recognized by TTH which considered it to be sufficient. The management of both hospitals granted permission for the interviews with healthcare professionals.

Prior to the interviews, all participants provided verbal informed consent, a method approved by the ethics committee in light of the social distancing measures and apprehension caused by the COVID-19 pandemic. To ensure participants fully comprehended the informed consent process, they were asked to confirm their understanding of the information provided, pose any questions they might have had, and give their permission to proceed with the interview. Consent to participate was implied through acceptance to proceed with the interview. Any potential participants who declined at this stage were respectfully excused from the process. All other principles stipulated in the Declaration of Helsinki were strictly adhered to throughout the study.^13^

## RESULTS

### Characteristics of the Respondents

The findings are organized around three major themes: COVID-19 intervention strategies, motivating and demotivating issues arising from response plans, and strategies and policies put in place to guide health workers caring for COVID-19 patients. We present the results in this manner because each policy, plan, or strategy was perceived by health workers to have had a distinct impact on health workers' motivation.

### The Predominant Themes

Outline in Table 1 is the triad of major themes derived from the current study, inclusive of their respective subthemes and codes. Simultaneously, Table 2 offers a comprehensive clarification of the response strategies that were implemented by the two countries under scrutiny.

**TABLE 1:** The Overarching Themes, Sub-themes and Codes

Theme	Sub-theme	Codes
COVID-19 Intervention strategies	Services delivery interventions	COVID-19 confirmed cases management Guidelines on COVID-19 testing Arrangement of COVID-19 duties
	Stewardship and leadership	Support supervision Psychosocial support
	Human resources for health interventions	Capacity building Motivation incentives declarations
Motivating Factors	Motivators related to services delivery interventions	Free COVID-19 testing
	Motivating factors related to the leadership and governance	Leadership and governance The psychosocial staff
	Motivation arising from financial incentives	The risk allowances
Demotivating Factors	Demotivators associated with service delivery interventions	Safety concerns Testing services delays Unsatisfactory handling of discharged patients
	Demotivating factors related to medical products and technologies	Inadequate logistic Harsh working conditions
	Demotivation related to the leadership and governance	Late response to the pandemic
	Demotivation related to human resources for health interventions	Frontline health worker definition Inadequate risk allowance Delayed payment of risk allowances Failure to honor some promised incentives Delayed training of frontline health workers

**TABLE 2:** Intervention Strategies Implemented by the Two Countries

Health Systems Building block	Intervention	Uganda	Ghana
Services Delivery	Management of confirmed and suspected cases	The Entebbe Regional Referral Hospital was designated as the primary facility for managing COVID-19 cases, which were identified both at the airport and within community settings. Consequently, this led to the suspension of non-COVID-19 services, encompassing all other routine health services, for an extended period of approximately one and a half years. In contrast, other public district & regional referral hospitals across the nation established designated COVID-19 treatment centers, primarily catering for cases necessitating intensive care. Subsequently, the management of non-critical COVID-19 patients was extended to all government health facilities, inclusive of national hospitals, regional and district hospitals, as well as Health Center IVs.^13^	The initial institutions designated for the management of COVID-19 cases included the Greater Accra Regional Hospital, Tema General Hospital, Korle-Bu Teaching Hospital (KBTH), Komfo Anokye Teaching Hospital (KATH) in Kumasi, the Greater Accra Regional Referral (RIDGE) Hospital, Ga Hospital, and the University of Ghana Medical Centre, in addition to all regional and training hospitals. Subsequently, the responsibility of COVID-19 management was extended to several other hospitals. In response to the lack of an infectious disease center in the northern region, a COVID-19 treatment center was established at the Tamale Teaching Hospital to provide care for individuals diagnosed with COVID-19.
	Guidelines and processes on COVID-19 testing	Initially (during the first two months of the pandemic), testing for all samples from COVID-19 suspects was limited to the Uganda Virus Research Institute (UVRI) for all samples collected in Uganda. More COVID-19 testing centers were later opened in other parts of the country, including Entebbe Regional Referral Hospital (ERRH).	The initial phase of COVID-19 testing was conducted at two primary institutions: the Noguchi Memorial Institute for Medical Research and the Kumasi Centre for Collaborative Research (KCCR). During this period, the average time to receive test results ranged from 48 to 72 hours. However, as the country expanded its testing capacity, the turnaround time for results was significantly reduced to less than 48 hours. This improvement was largely due to the introduction of additional testing centers across the nation, including a notable facility at the Tamale Teaching Hospital.
	Arrangement of duty for COVID-19 care	The frontlines Health workers were isolated from their families and stationed at the hospital. They were only granted permission to return to their families/homes only once in two weeks	COVID-19 frontline health workers commuted daily from their homes/residences.
Stewardship and Leadership	Support supervision	Ministry of health officials conducted regular support supervision at ERRH hospital for COVID-19 related service delivery. Ministry of health officials attended patient discharges, which was a morale booster for frontline health workers, according to a health worker at Entebbe hospital.	The president gave a weekly update on the COVID-19 status quo. At these weekly updates, he would pronounce new measures taken to combat the pandemic
	Psychosocial support & care	The Ministry of Health deployed psychologists and psychiatrists from Butabika hospital, the national mental health hospital to the hospitals to take care of the psychosocial and mental health of both health workers and patients.	Deployed later on in the course of the pandemic.
Human Resources for Health	Capacity building	A week of intensive training for the response team prior to the announcement of the first COVID-19 case.	Training of response teams conducted in Accra the capital dty.
	Declarations on monetary and non-monetary motivational incentives	A risk allowance for all FHWs, classified into three categories based on their risk of COVID-19 infection in the line of duty.^15^ The first group consists of high-risk frontline health workers who interact with people being treated for COVID-19 in Institution Treatment Units, such as doctors, nurses, clinicians, dispensers, and those in charge of general cleaning. These are paid 80,000 Ugandan Shillings (21 USD) per day of exposure or duty The moderate risk FHWs included laboratory personnel, clinicians assigned to monitor the signs and symptoms of COVID-19 suspects, and surveillance officers assigned to institutional quarantine centers to monitor contacts of people infected with COVID-19. Each day of duty was paid 60,000/= (16USD) Those deployed to make community follow up but are based at the institutional quarantine centre are paid 50,000 Ugandan Shillings (13.5USD) and include the drivers of ambulances, field clinicians and other staff. The low-risk FHW included the cooks and the gatemen who were paid 20,000/= (5.5USD).	All frontline health workers received a 50% basic monthly salary increase as a risk allowance. A medical officer and a graduate nurse, for example, who earned around 5000 and 2400 Ghana Cedis (900 and 430 US Dollars), respectively, were entitled to half of that for a month. Frontline health workers were exempted from paying income tax on their wages. Each frontline health worker was to receive a Ghana Cedis 350,000 (US$ 60,345) insurance package. Those who conducted contact tracing were paid 150 Ghana Cedis per day (26 dollars). The government promised to pay all water bills for all Ghanaians for the first three months beginning in April 2020, which was later extended for another three months. ^16^

### Motivating Factors Related to the COVID-19 Interventions and Response

During the pandemic period, the motivation of health workers in the two hospitals was affected similarly in some cases and differently in others.

### Motivating Factors Related to Service Delivery Free COVID-19 Testing

The provision of free COVID-19 testing, particularly for those suspected of infection and health workers potentially exposed in the line of duty, was a significant motivator. This was particularly evident in Uganda, where routine government services, inclusive of testing, were offered at no cost, contrasting with Ghana where such services necessitated possession of insurance. A respondent from Tamale Teaching Hospital in Ghana expressed profound gratitude for the government's decision to bear the financial burden of COVID-19 testing. This gesture was particularly impactful in the northern region of Ghana, characterized by a high population of low-income citizens, who would have otherwise been burdened by the prohibitive costs associated with testing:

*Yes, I believe that COVID-19 testing should be free of charge. That, in my opinion, is a good thing because not everyone could afford it, particularly in Northern Ghana, where money problems are worse than the exorbitant prices in Accra and Kumasi.* (Frontline Health worker 1, Tamale)

### Motivating Factors Related to the Leadership and Governance Provided during the Period of the Pandemic

#### Support Supervision

Health workers at the Entebbe Regional Referral Hospital (ERRH) were enthused by the robust support supervision from hospital administrators and officials from the Ministry of Health. This was seen as a crucial factor in enhancing the motivation and psychosocial well-being of health workers, thereby facilitating effective management of COVID-19. Moreover, during these supervisory visits, Ugandan government officials consistently acknowledged the frontline health workers as “Uganda's 2020 Heroes”. They proposed that these individuals should be awarded medals of honour in recognition of their invaluable services. Such affirmations served as a significant source of motivation, encouraging them to persist in their response efforts against the pandemic:

*… and I believe that whenever the Government of Uganda officials including those from the Ministry of Health came on board and we told them that we were discharging patients, the morale they gave us and what they kept telling us made us feel like they were on the frontlines psychologically with us. When we were handling our first case, the Prime Minister and the entire ministry were present. We got the impression that our service was highly valued.* (Manager, ERRH)

#### The Psychosocial Staff

In response to the COVID-19 pandemic, a contingent of mental health professionals, including psychologists, counselors, and psychiatrists, from the National Mental Health Referral Hospital were mobilized as part of multidisciplinary teams. Their primary role was to deliver psychosocial support to the healthcare workers who were assigned to manage COVID-19 patients at the hospital. The provision of this psychosocial care was both timely and crucial in mitigating the fear associated with the risks of COVID-19, thereby fostering mental stability among these frontline health workers. This information was corroborated by a medical officer at ERRH:

*We collaborated with Butabika hospital's mental health officers. They brought in psychosocial and psychological experts. These psychiatrists and psychotherapists came to see us at least once a week to check on employees who were having morale issues and to attend to their psychological needs. This was inspiring.* (Medical Officer, Uganda)

Mental health professionals, including psychiatrists and counselors, were deployed to communities with the objective of preparing residents psychologically for the reintegration of individuals who had undergone treatment for COVID-19. This initiative was met with gratitude by frontline health workers. However, the reception of this strategy varied between countries. In Uganda, it was largely well-received, whereas in Ghana, the late implementation of this approach led to less favorable reception. This is because by the time the mental health services were made available, many health workers were on the brink of succumbing to the psychological strain associated with caring for COVID-19 patients. Additionally, the number of clinical psychologists available in Ghana was insufficient to meet the high demand for their services.

### Motivation Arising from Financial Incentives in Form of Risk Allowance

#### The Risk Allowances

The financial incentives introduced by their respective governments provided an initial motivation for frontline health workers in both nations. The nature of these financial incentives varied between the two countries. A nurse from Ghana articulated the motivational atmosphere engendered by this approach as follows:

*I'll say that the tax break that we've all heard about has helped our morale to some extent. A military man does not return from battle empty-handed; he returns with a medal, assuming he is not killed on the battlefield. So those are our medals as well. I believe the Government of Ghana has done an excellent job putting together the package.* (Nurse, TTH)

It was suggested that the motivation driving certain Ugandan health professionals to join the COVID-19 frontline was purely due to the financial incentives associated with this role. This perspective was underscored by a respondent from ERRH, who implied that…

*Some health workers were ecstatic to be a part of the frontline response team because of the allowances. Due to the excitement of being chosen to be part of the response teams, it is believed that some of these frontline health workers paid little attention during the training and practical demonstrations on how to don and don-off personal protective equipment. This could have been the source of their irresponsible behavior, leading to COVID-19 infection.* (Hospital Manager, ERRH)

### Factors that Demotivated Frontline Health Workers

This section contains an analysis of the findings for factors that demotivated health workers.

### Demotivation Related to Services Delivery Interventions

#### Safety Concerns in Non-COVID-19 Wards

The health workers at Tamale Teaching Hospital expressed discontentment with the policy that mandated the allocation of specific units to manage COVID-19 cases. This policy also required the provision of safety measures, such as personal protective equipment, in these designated units, while other departments continued to deliver routine health services with minimal protection. The implementation of this policy was met with resistance as it was perceived to foster hazardous working conditions in wards that were not earmarked for COVID-19 management.

*As health workers in non-COVID-19 designated centers, we feel less protected, including having to attend to patients whose COVID-19 status we are unsure of due to the minimal protective measures provided. This is especially concerning given that many COVID-19 cases have been identified among patients admitted to the general ward for non-COVID-related reasons.* (Female Nurse, Tamale Teaching Hospital)

#### Delays in the Release of Test Results

The protracted process of administering diagnostic tests for patients suspected of COVID-19 infection adversely affected the morale of healthcare professionals. This delay not only extended the anticipated discharge date for patients but also, in some instances, led to congestion in hospitals. This was due to the fact that patient discharge was predicated on a negative test result, causing hospitals to retain a growing number of COVID-19 patients who were presumed to be on the path to recovery. The subsequent section presents the perspective of a medical officer from Ghana on this matter:

*I'm annoyed by the delay in releasing the test results. Assume I have a patient and am informed that the test results will be available in three days and later they delay. When the patient asks when they will be discharged based on the results, I tell them three days. However, some patients had to wait for close to ten days to be discharged since the results delayed. “What makes me think the patient will believe me?”* (Medical Officer Ghana)

Consequently, the delay in the release of results compelled certain healthcare professionals to resort to the dissemination of incomplete information or even falsehoods in an attempt to soothe their patients. This led to a significant erosion of trust in these frontline healthcare workers from the patients' perspective. The subsequent demoralization experienced by the healthcare workers was an inevitable outcome of this loss of trust:

*You know you owe the patient the truth. So, I relay information, then go back and realize it's incorrect, so at some point, I'm not going to give the patient good information, which eventually demotivates me.* (Medical Officer, Tamale Teaching Hospital)

#### Unsatisfactory Handling of Discharged COVID-19 Patients

The post-discharge management of patients, especially those of foreign origin and travelers, was found to be unsatisfactory, leading to a detrimental effect on the morale of the healthcare professionals who had previously provided care for these individuals. This was documented in the Ugandan case study:

*It is easy to become attached to COVID-19 patients when working on them. You'd like to follow them up to their homes after they've been released to see how they're doing, but that's nearly impossible. However, the best thing to do for such a patient is to give them the best possible care while they are in the hospital, as well as the most appropriate discharge. For example, there was a significant increase in the number of truck drivers. These drivers come from a variety of countries, including Kenya, Tanzania, Burundi, and South Sudan. You are not permitted to visit the South Sudanese community any longer. You could take them to the border and leave them there. It is none of our business what happens in South Sudan. We simply sent them back, and we had no idea what happened after. And now you're concerned about this patient, who had become a friend to you as a result of your interactions with them while you were caring for them.* (Nurse, ERRH)

### Demotivation Arising from Issues Relating to Medical Products and Technologies

#### Inadequate Logistics

A significant factor that undermined the motivation of healthcare professionals during the pandemic was the inadequate provision of personal protective equipment (PPE) in the general wards of Tamale Teaching Hospital (TTH). Despite the critical shortage of PPE, prioritization was accorded to Intensive Treatment Units (ITUs), thereby relegating healthcare workers in non-COVID-19 general wards to rely solely on face masks and aprons, both of which were scarcely available. The scarcity was so severe that masks were occasionally rationed, compelling nurses in the general ward to manage a week with merely three masks. This situation further exacerbated the demoralization among healthcare workers, as articulated by a nurse at Tamale Teaching Hospital:

*“It is said in Ghanaian culture that if your mother is in the kitchen, you will not go hungry. Similarly, in the treatment centers, we are the children with mothers in the kitchen.” (COVID-19 patients in COVID-19 treatment units).*

*“We are the first to receive this protective gear. The challenge has been for the other wards, and you will notice that there is community spread, and a positive case can be identified from any part of the hospital, in fact from anywhere in Ghana”.* (Frontline Nurse Tamale Teaching Hospital)

Sometimes, there was a surge in the incidence of patients requiring COVID-19 treatment in Intensive Therapy Units (ITUs). This necessitated an increase in sanitation measures to a minimum of thrice daily, despite the glaring inadequacy of protective gear. Management's attempts to placate the workforce by highlighting the global scarcity of personal protective equipment (PPE) were largely met with skepticism and dissatisfaction among the majority of health workers in Ghana. For example, health workers reasoned that there was a conspicuous absence of these supplies in hospitals despite numerous donations of PPE from philanthropic individuals and corporations, as reported by a frontline healthcare worker,

*Then we hear about people donating personal protective equipment at all hours of the day and night, and when you ask for it and they say it isn't there, you wonder where they go. Someone should tell us how much you earned and how much you donated. Then we'll know you don't have. But if you don't tell us how much you received, how much you spent, and on whom you spent it, I'll become agitated and demand for it.* (Medical Officer, TTH)

#### Harsh Working Conditions

The treatment facilities for COVID-19 at Tamale Teaching Hospital (TTH) were not equipped with air conditioning systems, resulting in elevated temperatures that made the working environment uncomfortable. The necessity for health workers to wear Personal Protective Equipment (PPE) in these hot conditions exacerbated the discomfort. A health worker on the frontlines expressed this sentiment by stating,

*If you put on the cover all, the entire protective clothing from head to toe, and enter the patients' rooms, which have only fans and windows, no air conditioning, you will sweat and stress if you stay in there for an extended period of time. You'll be completely exhausted by the time you get out of it and out of the coverall.* (Medical Officer, TTH)

Confronted with these obstacles, healthcare professionals were compelled to modify their methodology in managing COVID-19 patients. A portion of these professionals reported adopting a strategy of expedited care for COVID-19 patients with the aim of minimizing the duration spent in protective coveralls. Conversely, others opted to conduct patient assessments via telecommunication methods, thereby circumventing the necessity of direct interaction while donned in protective coveralls:

*However, when I enter and begin reviewing the patients, I sometimes have to rush and end up doing incomplete work; and sometimes I try to talk to the patients on the phone from time to time.* (Medical Officer, TTH)

#### High Workload

In both medical facilities, it was frequently reported that healthcare professionals were experiencing excessive workloads. The case study in Uganda revealed that the work overload was attributable to the limited number of healthcare workers initially recruited as frontline staff. However, as the number of cases escalated, thereby increasing the workload, the necessity for additional staffing became evident. Moreover, the transition from a traditional eight-hour, five-day work week to a more demanding twelve-hour, five-day schedule resulted in longer working hours, leading to strenuous work schedules.

In Ghana, the number of healthcare workers assigned to each shift had to be rationed to minimize the number of staff at risk during a specific time period. Consequently, when staff members on a particular shift were exposed and subsequently quarantined, this created staffing shortages, compelling the remaining nurses to assume additional responsibilities. In some instances, certain wards had to be temporarily closed due to insufficient staffing levels, as noted by one staff member:

*I recall taking some precautions to reduce risk in the first two cases we received in Tamale, such as allowing a few people to work in a week so that if an incident occurs, we can care for those people. We had no choice but to close the ward. Everyone who was on duty when the first COVID-19 cases arrived had to be quarantined, resulting in the loss of two-thirds of our human resources. Those of us who were not on duty at the time were forced to complete all of the work, which was a difficult task,* (Female Nurse, TTH)

### Low Morale as a Result of Leadership and Governance Issues

Health workers reported a decline in motivation during the COVID-19 pandemic, which they attributed to deficiencies in leadership and governance. These issues were particularly pronounced in relation to the delayed leadership pronounced response to the pandemic.

#### Late Response to the Pandemic

Certain healthcare professionals in Ghana expressed consternation regarding the perceived delayed reaction of their leadership to the COVID-19 pandemic. These medical practitioners posited that a proactive preparedness strategy could have facilitated a more efficient initial response to the health crisis. Regrettably, the nation's preparedness measures were only initiated upon the confirmation of COVID-19 cases within its borders. Consequently, officials were compelled to hastily procure supplies in a market already dominated by preemptive purchases from other nations. This situation inevitably led to an escalated cost in the nation's response to the pandemic.

*We had a chance to get our house in order before the pandemic, but I believe we squandered it. I believe it took about three months from the time COVID-19 was first mentioned in China in late 2019 until Ghana received its first case. Thus, Ghana began preparing for such a pandemic about three months late!* (Medical officer Tamale Teaching Hospital)

The health workers (HWs) posited that the government's delayed response resulted in suboptimal implementation of critical decisions, such as the timing and execution of the lockdown. They argue that the efficacy of Ghana's COVID-19 response could have been significantly enhanced had the decision-making process and preparatory measures for the pandemic been expedited.

### Demotivation Related to Human Resources for Health Interventions

#### Delayed Training of Frontline Health Workers

In Ghana, there was a unanimous agreement that the nation's preparedness, particularly in terms of training, was insufficient to effectively respond to the initial cases of COVID-19 when the pandemic was declared. Consequently, the inaugural training of trainers in Accra was expedited. The looming lockdown in Accra significantly affected the focus of the trainees. A frontline healthcare professional at the Tamale Teaching Hospital expressed dissatisfaction with this situation:

*We would have taken advantage of what was going on elsewhere and begun training as soon as possible, knowing that COVID-19 would arrive as soon as possible based on what we had seen elsewhere and the constant in-and-out travel patterns. We had just finished the trainer of trainees training in Accra when a lockdown was declared. Those of us who had attended the training and were planning on returning upcountry to prepare teams for our hospitals were almost caught up in the Accra lockdown.* (Emergency HCW, TTH)

#### The Dilemma of Defining Frontline Health Workers

In Uganda, the classification and definition of frontline health workers were clearly delineated, with their associated risk benefits explicitly outlined. This, however, was not initially the case in Ghana. There was a notable contention regarding the criteria for qualifying as a frontline health worker, particularly in relation to eligibility for government incentives such as a three-month tax exemption and a 50% salary increase as a risk allowance.

This discrepancy in the definition led to widespread dissatisfaction among the majority of healthcare providers, igniting debates among various health worker groups. Interestingly, even administrative personnel in hospitals, whose services had been temporarily suspended, were included in the initial definition of frontline health workers. This inclusion further fueled dissent among other medical professionals.

*By allowing all health workers to apply, the tax breaks and the announcement of a 50% salary increase for frontline health workers have exacerbated the motivation situation. We assumed you made that announcement to encourage workers who were more likely to contract COVID-19, but under this arrangement, many of them do not qualify.* (Male Frontline health worker, TTH)

In the aforementioned arrangement, individuals who perceived themselves as being at a higher risk felt that their administrative counterparts would gain advantages at their detriment.

Despite the fact that the definition of a frontline health worker was subsequently refined to encompass only those serving in COVID-19 Isolation Treatment Units (ITUs), a sense of exclusion and dissatisfaction was reported among some healthcare professionals. Those discontented with the ultimate categorization of frontline health workers provided evidence that several of their colleagues had contracted the virus outside of the COVID-19 ITUs. Furthermore, there were instances of health workers who had been quarantined following exposure in general wards, yet the revised definition precluded them from being recognized as frontline health workers due to their non-affiliation with the COVID-19 ITUs. This sentiment of exclusion is encapsulated in the following excerpt from a frontline health worker:

*We only have a group of 25 nurses in this large institution of Tamale Teaching Hospital who are most likely frontline nurses, according to the definition of a frontline health worker being those working in ITUs. What about the other nearly 1500 nurses who could have been infected with the virus at any point in the hospital?* (Nurse TTH)

Consequently, it was observed that certain health workers not assigned to COVID-19 isolation units, occasionally expressed a sense of non-obligation towards providing medical care to individuals suspected of being infected with COVID-19 in the general wards. This perception stemmed from the assumption that since they were not from the category of frontline healthcare workers by virtue of not working in the isolation units, the responsibility of attending to such patients was designated to other personnel given their roles in the isolation units who were specifically remunerated for this task.

*There is this patient on the ward who is suspected of having COVID-19 symptoms or signs, but the non-frontline health worker chooses not to attend to him. The critical care that the patient should have received is delayed by summoning a frontline health worker to attend to him.* (Medical Officer, TTH).

#### Inadequate Risk Allowance

Healthcare professionals in Uganda expressed dissatisfaction with the inadequate risk allowance provided to them. As per the perspective of a hospital administrator, “*A risk allowance is indeed allocated to them. While some of these funds are remitted to their families, the amount is insufficient to sustain a household*.” (Hospital Manager, ERRH).

The dissatisfaction among healthcare workers eventually led to a diminished interest in their roles as frontline COVID-19 healthcare providers. It was reported that some of these health workers paid inadequate attention to training sessions and practical demonstrations on the usage of personal protective equipment, ostensibly due to insufficient allowances. This lack of focus is believed to have resulted in a deficiency in their skill sets. Consequently, this is postulated to have played a significant role in the high incidence of hospital-acquired COVID-19 infections.

#### Late Release of the Risk Allowance

The delayed disbursement of the anticipated risk allowances elicited expressions of dissatisfaction and demoralization among healthcare professionals in both nations. This delay was perceived by the healthcare workers as a sign of disrespect and a lack of recognition for their unwavering commitment to their respective countries. This sentiment was echoed by a frontline worker in Ghana,

*“Obviously, I would not be concerned if I had not expected it. I'd just go about my business, but having heard well that this is something you're going to get and it's not coming' dampens my spirits a little”.* (Frontline health worker, Ghana)

As per the testimony of a manager at Entebbe Hospital, some health workers exhibited a diminished enthusiasm towards their roles as frontline health workers primarily attributed to the postponement in the disbursement of allowances due to them.

*“The anticipated risk allowance paid to frontline health workers fueled many frontline health workers' enthusiasms for joining COVID-19 treatment teams. However, after waiting and waiting for it to arrive, some of the workers began to complain. Morale eventually plummeted”.* (Manager, Entebbe hospital)

Due to the delay in the disbursement of the risk allowance, a number of health workers were under the impression that they had been deceived and would not be the recipients of the said allowance. This situation evoked memories of past encounters with regular programs where the allocation of funds was characteristically delayed and eventually missed out altogether.

*“As a medical officer providing services at the COVID-19 quarantining center, I have heard staff members express dissatisfaction with the situation and feel duped by the Ghanaian government”.* (Medical Officer, TTH).

Another nurse noted that;

*“People were very excited with the allowance and believed that our struggle was being understood, but I don't know anyone who has received the salary increment. I've seen the frustration. A few of my coworkers have also expressed dissatisfaction, primarily because they believe they have been betrayed or lied to as a result of the presidential proclamation to raise the pay of frontline workers, which has yet to be implemented”.* [Nurse frontline health worker, TTH].

#### Failure to Honor Other Motivational Incentives

In addition to the inherent shortcomings associated with risk allowances and other reliefs, healthcare professionals have expressed a sense of demotivation stemming from unfulfilled commitments. A number of healthcare workers in Ghana were enticed to participate in the COVID-19 response teams through assurances of accommodation and transportation, which unfortunately were not upheld. In instances where these healthcare workers contracted COVID-19 and required confinement in isolation centers specifically designed for exposed health workers, there were no established transportation arrangements for them. Consequently, they were compelled to utilize public transportation. According to a frontline health worker at the Tamale Teaching Hospital (TTH), this situation not only posed a risk to the public but also served as a source of demotivation.

*For example, when a group of us from this unit became infected with COVID-19 and were summoned to the quarantine center, I expected the hospital to arrange transportation for us to be quarantined, as they had promised us during orientation. We took public transportation to the quarantine centers because no one cared about us. I believe the hospital could have performed better.* (Frontline nurse, TTH)

#### Health Workers' Families are not Taken into Account

Certain frontline health workers, particularly those engaged in Entebbe Regional Referral Hospital in Uganda, voiced apprehensions regarding the wellbeing of their families while they were confined as they cared after COVID-19 patients. They expressed that in their absence, their dependents may have been experiencing food scarcity and that their children may not have been receiving sufficient medical attention. Regrettably, the motivational package provided to these healthcare workers did not address the needs of their families. This oversight left the children of these professionals in a state of uncertainty, unaware of their parents' whereabouts or the nature of their work. Consequently, as articulated by a respondent from Entebbe Hospital, this neglect towards the familial responsibilities of healthcare workers served as a demotivating factor:

*Some frontline health workers, particularly those involved in ERRH in Uganda, expressed concern about their families going hungry and their children not receiving adequate care when they became ill. Unfortunately, the motivation package made no mention of reaching out to health workers' families or what happens to them. Some children were unaware of what was going on while their parents were away. As a result, as one respondent in Entebbe Hospital summarized, such healthcare workers were demotivated.* (Manager Entebbe Hospital)

For health workers infected with COVID-19, the situation got worse because their families back home received no material or psychosocial care. *Another issue that has not been addressed is that, in addition to isolating and keeping infected health workers in the hospital, we have not visited their families. However, if I keep this healthworker, I believe the ministry should consider how to assist their families and anything else that comes with that package. This would be beneficial.* (Hospital Manager, Entebbe Hospital)

#### Quarantined Health Workers Poorly Managed

Some TTH health workers were dissatisfied with the treatment of quarantined health workers. For example, health workers believed that hospital administrators should have contacted them to inquire about the well-being of their family members

*I had the impression that we were not being treated well even while we were in quarantine. I was hoping for more. I expected managers to contact me to inquire about my family's well-being, because if my family is not stable, neither am I.* (Nurse, TTH)

Even after such health workers were discharged, nothing was given to them to help them care for their families, despite the fact that they would have been quarantined and would have to stay at home for some time to avoid stigma from the community. As a result, an Entebbe hospital respondent proposed the following.

*“In the event of quarantined health workers suspected of contracting COVID-19, supervisors should have maintained regular communication with their families, provided emotional support, offered practical assistance, monitored their health, and provided financial aid. Additionally, managers should have maintained contact with discharged health workers to boost morale and alleviate post-discharge stigma and anxiety.”* (Hospital Manager, ERRH).

## DISCUSSION

This study critically and deeply investigated how the initial COVID-19 pandemic response strategies were perceived in relation to the motivation of frontline health workers caring for COVID-19 patients.

### The Motivating Strategies

The free provision of services to COVID-19, health workers' training, support supervision, and good management and leadership demonstrated by the MoH and hospital officials were cited as the most motivating factors by health workers. Previous research has also found that the top motivators in a pandemic response include training, support supervision, especially for infection control, and transformative leadership.^14^ It has been stated that comprehensive support supervision for frontline health-care providers is critical in responding to COVID-19, particularly to supervise infection prevention and control measures.^15,16^ During the Ebola outbreak in West Africa, it was discovered that support supervision was critical for building resilient health systems to respond to the outbreak.^17^

Training, on the other hand, was critical in the response to the COVID-19 pandemic because it taught health workers how to avoid contracting the disease and alleviated their anxiety about the disease's unknown management methods.^18^ However, the manner in which it was conducted and implemented demotivated some HCWs, as evidenced in Ghana, in particular, where the training was perceived to have been conducted late and hurriedly. This challenge of hurriedly organized training was echoed in other studies in sub-Sahara Africa. ^19^ Rapidly training healthcare professionals for a viral epidemic, such as COVID-19, could lead to inadequate knowledge, compromised skills, elevated risk of mistakes, and heightened stress levels. ^20^ These factors may have adversely affected patient care and the overall effectiveness of the response.

### Demotivating Factors Related to the COVID-19 Interventions

Despite the implementation of numerous interventions in response to the COVID-19 pandemic, healthcare workers (HCWs) experienced demotivation due to the way the planning and execution of these measures were done. The pandemic revealed deficiencies in outbreak preparedness, including difficulties in allocating risk allowances and other incentives, safety concerns, and delays in testing services. Furthermore, logistical support was insufficient, and patient discharge management was suboptimal. The working conditions were exacerbated by the necessity to convert rooms with inadequate air conditioning into intensive care units, particularly in the TTH. The process of identifying frontline health workers and the delay in their training were additional human resources for health interventions that contributed to the demotivation.

The identified gaps in pandemic preparedness highlight deficiencies within the national healthcare systems and preparedness policies of both countries that have also been raised in other health systems in Africa. ^21^ Insufficient resources may subsequently hinder healthcare workers' ability to effectively deliver response services as seen here and in other studies. ^22^ Manifestations like these diminish the confidence and motivation of health workers in delivering services during outbreaks of the magnitude of COVID-19, underscoring the necessity for enhanced preparedness in future response endeavors. These findings further emphasize the importance of formulating policies and motivational interventions that are acceptable to the staff who will be affected by these strategies. This approach is crucial to ensure the effectiveness of such interventions and to maintain the motivation and morale of healthcare workers during challenging times. ^23^

The delays in releasing testing results were primarily due to insufficient testing capacity, as neither country had adequately prepared for the COVID-19 testing. This had a major impact on service delivery. In Ghana, the treatment and patient discharge protocols were revised, and the country deviated from the WHO guideline for patient discharge to avoid problems associated with delayed patient discharge due to results delays.^20^ As the number of COVID-19 cases increased in Uganda, for example, government laboratories' testing capacity became overburdened, necessitating the involvement of testing in for-profit private health facilities. However, this came at a cost of approximately $65, of which many members of the general public could not afford.^21^ Patient management was severely hampered as a result, leading to demotivation among frontline HCWs. A humanistic care approach should be fundamental in the COVID-19 and any other pandemic response, particularly at the time of discharge when patients are unsure of the course of their illness.^22^

Frontline HCWs in TTH complained about a hostile work environment, especially the extremely hot ITU rooms where COVID-19 patients were isolated for management. Engineering controls rank first in controlling hazards, particularly those related to influenzas, an issue which seems not to have been fully addressed in this case.^24^ The International Labor Organization also highlights the importance of having proper working conditions for caregivers of COVID-19 patients.^24^

In both countries, the planning of motivational incentives encountered difficulties, culminating in the demotivation of health workers. The delayed disbursement of risk allowances in both countries raised questions about the likelihood of HCWs receiving these benefits, subsequently leading to diminished employee morale. Media reports indicated that health workers in various Ugandan hospitals, inclusive of the primary testing center, initiated strikes due to the tardiness in allowance payments.^25^ The issue of delayed allowance disbursement, resulting in HCW demotivation, has been a prevalent challenge in Sub-Saharan hospitals during the COVID-19 pandemic response.^24,26^

Risk allowances and other incentives necessitate meticulous planning and management to stimulate employee motivation during the COVID-19 pandemic and future pandemics. This includes the clear communication of the purpose and methods for allocating the risk allowance to HCWs^27^. To prevent confusion and strikes, it is imperative that the incentives are distributed to the intended recipients promptly and as per the agreed schedule. However, this is only effective if the communicated welfare plans are deemed suitable and acceptable by the health workers, following their consultation and approval of the plans in a systematic manner. In the pandemic context, the principle of equity should supersede education level or seniority in the implementation of monetary motivational incentives. In this regard, risk exposure should be the primary determinant in the allocation of monetary motivation incentives, rather than the level of education.^28^

## CONCLUSION

The most important lessons learned from the COVID-19 pandemic is the importance of being prepared for unforeseen outbreaks and disasters putting into consideration the effect of the response preparedness on health workers motivation and morale. In the event that an entire array of incentives does not become achievable, this may lead to demotivation of healthcare workers affecting pandemic or outbreak responses as observed with the response strategies during COVID-19 in this study. It is essential that governments learn these lessons to plan better responses so as health workers are stimulated to respond to outbreaks of COVID-19's magnitude better. Health workers' opinions should be sought while designing specific response strategies and how best to apply them during outbreak. This approach will most likely ensure a comprehensive and effective response to health outbreaks in the future.
